# A (Permalloy + NiZn Ferrite) Moldable Magnetic Composite for Heterogeneous Integration of Power Electronics

**DOI:** 10.3390/ma12121999

**Published:** 2019-06-22

**Authors:** Chao Ding, Yunhui Mei, Khai D. T. Ngo, Guoquan Lu

**Affiliations:** 1 The Department of Materials Science and Engineering, Virginia Tech, Blacksburg, VA 24061, USA; dingchao@vt.edu; 2 School of Materials Science and Engineering, Tianjin University, Tianjin 300072, China; yunhui@tju.edu.cn; 3 The Bradley Department of Electrical and Computer Engineering, Virginia Tech, Blacksburg, VA 24061, USA; kdtn@vt.edu

**Keywords:** heterogeneous integration of power magnetics, soft magnetic moldable composite (SM^2^C), Permalloy + NiZn ferrite powdered core, low-temperature and pressure-less processing

## Abstract

Soft magnetic moldable composites (SM^2^Cs) would be ideally suited for the integration of magnetic components in power electronic converters because they can be formed into magnetic cores by low-temperature and pressure-less processing. However, most SM^2^Cs have low relative magnetic permeability, typically less than 30, and high core-loss densities at switching frequencies over 1 MHz. To improve their magnetic properties, we combine powders of Permalloy and a NiZn ferrite with an acrylic polymer to formulate a paste of SM^2^C. The paste can be molded and then cured below 200 °C without pressure to form cores with a relative permeability over 35 and a core-loss density at 1 MHz, 30% lower than those of commercial cores. The ease of its processing and high-performance properties makes the SM^2^C a good candidate material for the integration of power magnetics.

## 1. Introduction

Integration of magnetic components in the manufacturing of power electronic converters has the potential to increase converter power density and efficiency at a lower cost [1,2,3,4]. Due to their low-temperature and pressure-less processability, soft magnetic moldable composites (SM^2^Cs) would be the materials of choice to simplify the integration effort [5,6,7,8]. SM^2^Cs consist of magnetic powders in a polymer that can be formed into various shapes by molding or casting and curing at a low temperature without external compaction pressure. The fabrication process is compatible with traditional printed circuit board (PCB) manufacturing or emerging additive manufacturing to enable co-fabrication of windings and other components [8,9]. For example, one may envision using the magnetic material together with metal and insulation materials in a multi-extrusion 3D printer to fabricate a converter’s passive elements and interconnect all of the printer platform [10]. However, to realize this vision, magnetic properties of the existing SM^2^Cs have to be improved to have relative permeabilities over 30 and core-loss densities comparable to commercial iron-power cores for switching frequencies into the MHz range.

Many formulations of SM^2^Cs have been reported in the literature. In [11,12,13,14,15,16,17], ferrimagnetic materials (NiZn or MnZn ferrite) were used as magnetic fillers. The reported relative permeabilities of these materials range from 6 to 25 depending on the filler’s composition, shape, particle size, size distribution, and the amount added. In [8,18,19,20,21], ferromagnetic materials (iron or iron-based alloys) were explored as fillers, and the achieved relative permeabilities ranged from 3.5 to 20.5. In our previous work [22], a low temperature-curable SM^2^C was developed with the guidance of Finite Elements Analysis by ANSYS Maxwell. Instead of only using spherical powders in the formulation, flake-shaped powders made by crushing a Metglas amorphous alloy ribbon were added to create “conductive” paths for magnetic flux and thus increased the magnetic permeability. A maximum relative permeability of 26 was achieved with 12.5 wt% of Metglas flakes in the total amount of the magnetic fillers. However, the core-loss density was high at a frequency over 1 MHz because of the low electrical resistivity of Metglas flakes resulting in high eddy-current loss.

In this work, we replace the electrically conductive Metglas flakes with sintered NiZn ferrite flakes to increase relative permeability and reduce core-loss density. Ferrite flakes provide high “conductive” paths for magnetic flux but also high electrical resistivity to reduce eddy current loss at high frequency. The other magnetic filler, a Permalloy powder, was the same as what was used by Yan et al. [22]. We modify the organic system used in the previous work to decrease the processing temperature to 200 °C and increase packing density by eliminating trapped air bubbles. The improved SM^2^C formulation is processed without pressure by pouring the paste-form material into a toroid mold, followed by curing. The relative permeability dispersion plots, core loss densities, hysteresis loops, and direct current (DC) characteristics are measured. Microstructures of the cores are characterized by scanning electron microscopy (SEM).

## 2. Experimental Procedure

### 2.1. Material Selection

In Yan et al.’s work [22], they formulated a SM^2^C (also called magnetic paste) by mixing Benzocyclobutene (BCB) with magnetic fillers. BCB is expensive and requires curing at over 250 °C for 1–2 h. Their paste also had high viscosity and was easy to trap air bubbles in the core, which decreased magnetic permeability. In this work, we replaced BCB with Trimethylolpropane triacrylate (TMPTA) (from Sigma-Aldrich Co. LLC, St. Louis, MI, USA) monomer solution. The monomer solution is low cost, has a viscosity similar to water, and polymerizes at less than 200 °C with minimal production of by-products.

As for magnetic fillers, Permalloy and Metglas powders were selected in the previous study because of their high initial permeabilities, low hysteresis losses, and high-saturation magnetization. In this study, we replaced the Metglas flake powder with a sintered NiZn ferrite flake powder to take advantage of the high electrical resistivity of the ferrite material. We acquired two kinds of sintered ferrite material, one from Powdertech International Co. (Valparaiso, IN, USA) with an average particle size of 150 μm and the other from 3M, one of 3M™ Flux Field Directional (St. Paul, MN, USA) sheet materials. The 3M sheets were crushed and sieved to generate a flake-shaped powder with an average particle size of 500 μm. The second filler material was kept the same as that was used by Yan et al., i.e., a Permalloy powder (from ESPI Metals, Ashland, OR, USA) with an average particle size of 12 μm.

### 2.2. Preparation of SM^2^C

**Figure 1:**
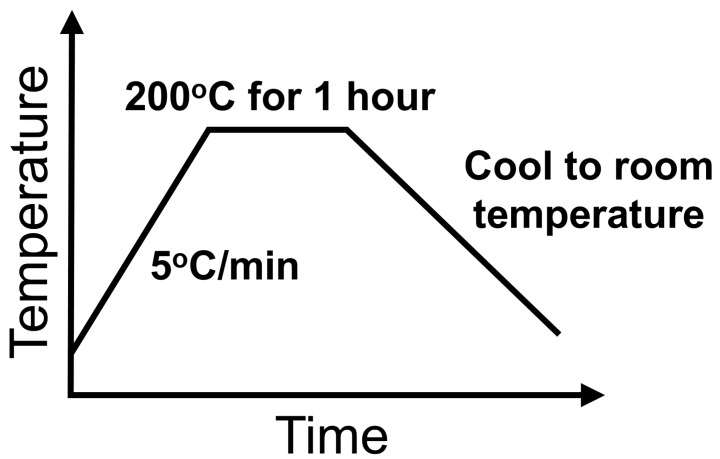
The heating profile used to turn the paste formulations into solid SM^2^C cores.

Two formulations of SM^2^C were prepared by mixing TMPTA with Permalloy powder and one of the two ferrite powders. In both formulations, we kept the polymer content the same at 5 wt% for bonding and insulating the magnetic filler particles. In Formulation A, the amounts of filler powders added were 47.5 wt% of Permalloy and 47.5 wt% of sintered NiZn ferrite flakes from Powdertech International Co.; in Formulation B, the fillers were 66.5 wt% of Permalloy and 28.5 wt% of the 3M flakes. Table 1 lists the compositions of the two formulations. To ensure mold-ability and flow-ability of the pastes, small amounts of organic surfactant and solvent were also added. The ingredients of each formulation were mixed by hand in a plastic jar and then homogenized after shaking for 10 min in a high-speed vibrating ball miller (MSK-SFM-3, MTI Corporation, Richmond, CA, USA). The smooth magnetic pastes flowed easily, filling corners and cavities in the molds. To solidify the content in the mold, heating was used to cure or polymerize the polymer resulting in a solid SM^2^C core. The heating profile is shown in Figure 1. A ramp rate of 5 °C/min was used to slowly evaporate solvents and avoid bubbles trapped inside. The paste was soaked at 200 °C for one hour to fully solidify.

### 2.3. Characterization

All the samples used to characterize properties of our formulated materials in this study were made by a molding process. The SM^2^Cs in paste form were poured into molds with desired dimensions, followed by curing at 200 °C for one hour without external pressure. After demolding, the samples with desired dimensions were obtained.

To characterize permeabilities, core-loss densities, and DC characteristics of our SM^2^C formulations, toroid cores were made using the process described above. The cores’ relative permeabilities versus frequency were measured using a precision impedance analyzer (4294A; Agilent, Santa Clara, CA, USA) with a magnetic material test fixture (16454A; Agilent, Santa Clara, CA, USA). The core-loss densities versus B_peak_ at 1 MHz were measured using a custom-designed setup described in [23,24,25]. To measure the DC bias permeability of a toroid core, two copper windings were wrapped around the core. One of the windings was used to flow a DC current introducing a DC magnetic field into the core, while the relative permeability of the core was determined by inductance measurement using the other winding. The inductance was measured by a precision impedance analyzer (4294A; Agilent, Santa Clara, CA, USA). To characterize the magnetic hysteresis loops, cuboid samples with dimensions of 5 mm × 3 mm × 1 mm were also made using the processes described above. The samples were tested in a Physical Property Measurement System (PPMS-9, Quantum Design, San Diego, CA, USA) with a maximum magnetic field of 800,000 A/m. The microstructures of cores made from Formulations A and B were characterized by scanning electron microscopy (SEM, LEO 1550, Zeiss, Jena, Germany).

## 3. Results and Discussion

### 3.1. Relative Permeability versus Frequency

**Figure 2:**
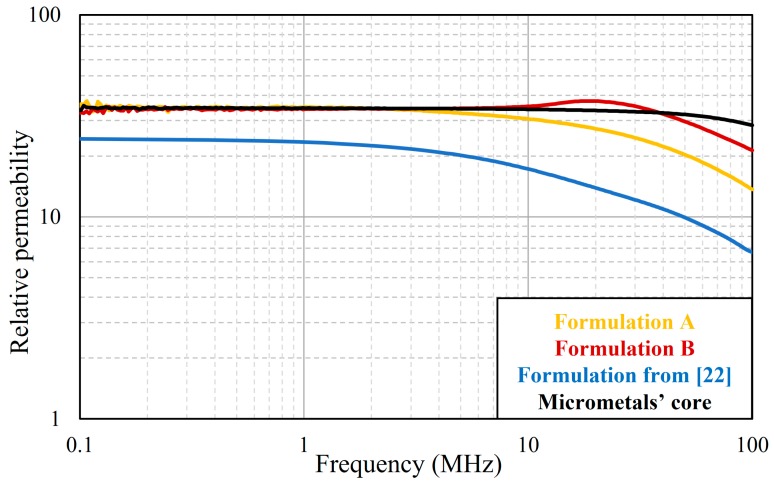
Plots of relative permeability versus frequency of Formulations A and B, a formulation from [22], and that of a commercial core from Micrometals.

Shown in Figure 2 are plots of the relative permeability versus frequency of cores made from the SM^2^C Formulations A and B and a formulation from [22]. For comparison, we also measured the dispersion curve of a commercial iron-powder core (Power Conversion Materials-8, Micrometals, Anaheim, CA, USA) with similar dimensions. The commercial core requires high compaction pressure (usually several hundred MPa) to make. The relative permeabilities of both of our current formulations exceeded 35, which is 35% higher than that of our previous formulation of 26. This is because we replaced Metglas flakes with sintered NiZn ferrite flakes and added more fraction of ferrite flakes. Our improved SM^2^Cs have similar relative permeabilities to that of the Micrometals’s core up to 3 MHz. The Micrometals’s core has a steady relative permeability up to 10 MHz, while that of Formulation A started to drop after 3 MHz and Formulation B after 40 MHz. On the plot for Formulation B, there is a broad hump at around 20 MHz. We believe that this was caused by the magnetic resonance [26,27] of the ferrite particles in the composite. This resonance phenomenon is common in ferrite materials although the humps in single-phase ferrites are normally sharper than what we observed. Formulation B was a multi-phase material with 32 vol% ferrite and the rest made up of Permalloy and an organic binder. We speculate that the presence of Permalloy in the composite might have broadened the resonance of the ferrite. However, the plot for Formulation A has no hump even though it also contained ferrite particles. We do not have an explanation for the missing hump in Formulation A except to note that the ferrite flakes used in A and B are from two companies and have different chemistries. It is well known that the resonance phenomenon in ferrite depends strongly on composition and processing conditions, such as sintering temperature, atmosphere, and pressure. Overall, the addition of the sintered NiZn ferrite flakes increased the relative permeability of our SM^2^Cs over 35, which is the highest relative permeability obtained in the reported SM^2^Cs.

### 3.2. Core-Loss Density

**Figure 3:**
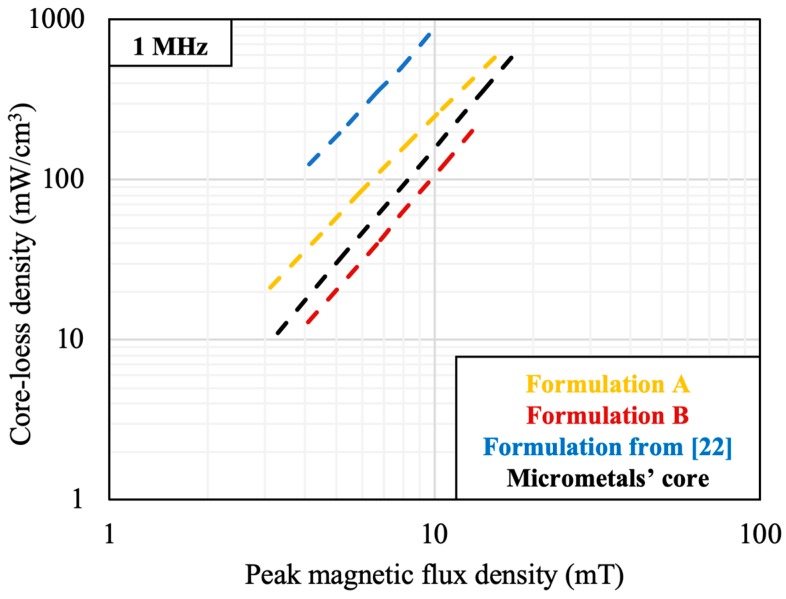
Plots of core loss density versus magnetic flux density at 1 MHz of Formulations A and B, a formulation from [22], and that of a Power Conversion Material-8 core from Micrometals.

Figure 3 shows the core-loss densities of magnetic cores made with Formulations A and B, a formulation in [22], and one from Micrometals. The four formulations were tested using the same setup and under the same conditions at 1 MHz and room temperature. Formulations A and B have respectively 72% and 89% lower core-loss density compared to the formulation from [22]. Replacing Metglas flakes with sintered NiZn ferrite flakes not only increased the relative permeability but also decreased the core-loss density because ferrite has a much higher electrical resistivity than that of Metglas 2705M (Co-based amorphous alloy, Metglas, Conway, SC, USA), for reduced eddy current loss at high frequency. At 1 MHz, Formulation A has a 67% higher core-loss density while Formulation B is 33% lower than that of the commercial core from Micrometals (Anaheim, CA, USA).

### 3.3. Magnetic Hysteresis Loop and Saturation Magnetic Flux Density

**Figure 4:**
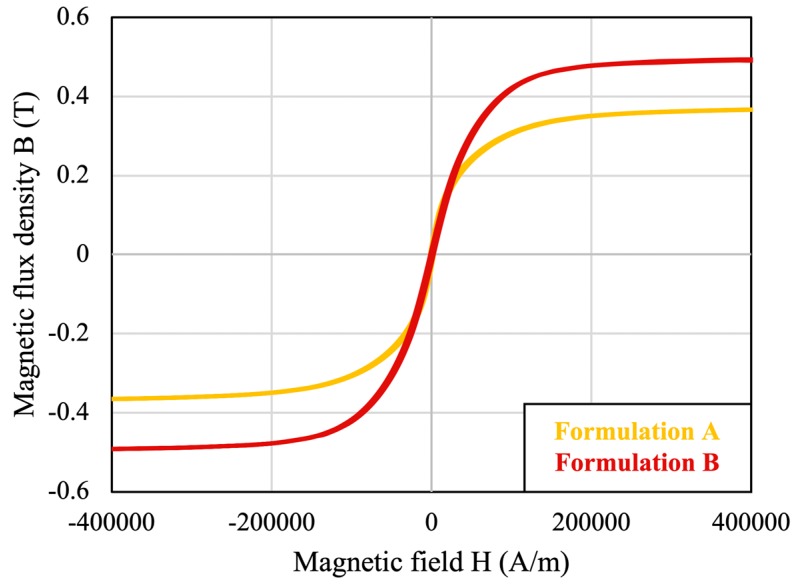
B-H curves measured on Formulations A and B.

Figure 4 shows the magnetic hysteresis loops, or plots of flux density (B) versus magnetic field (H) measured on Formulations A and B. The saturation flux density, *B_sat_*, is the point on the B-H curve at which the slope of the curve equals to vacuum permeability (µ_0_) [28]. From Figure 4 the values of *B_sat_* of Formulations A and B are 0.36 T and 0.49 T, respectively. Formulation B has a higher *B_sat_* because it has higher ferromagnetic content as shown in Table 1.

Sung and Rudowicz [29] offered a simple model for estimating the *B_sat_* of a SM^2^C. The magnetization of a material is defined as the magnetic moment per unit volume. For a composite consisting of a number of magnetic fillers,

(1) $$M=\frac{1}{V}\sum m_{i}$$

where *M* is the magnetization, *V* is the volume, and *m_i_* is the magnetic moment of *i*th filler in the composite. Applying the mixing rule, the saturation magnetization of a SM^2^C can be written as:

(2) $$M_{s}=\frac{1}{V}\sum M_{si}\cdot V_{i}=\sum M_{si}\cdot \varphi_{i}$$

where *M_s_* is the saturation magnetization, *M_si_* is the saturation magnetization of the *i*th filler, and $\varphi_{i}$ is the volume fraction of the *i*th filler. The relationship between magnetic flux density (*B*) and magnetization (*M*) is $B=\mu_{0}\cdot \left(H+M\right)=\mu_{0}\cdot \left(H+\chi_{m}\cdot H\right)$ in SI unit, where $\chi_{m}$ is the magnetic susceptibility of the composite. For a ferro- or ferrimagnetic material, *M* is much larger than *H* since $\chi_{m}$ is much larger than 1. Thus, the B_s_ of a SM^2^C can be approximated by [30]:

(3) $$B_{sat}=\sum B_{si}\cdot \varphi_{i}$$

where *B_sat_* is the saturation flux density of the SM^2^C, *B_si_* is the saturation flux density of the *i*th filler, and $\varphi_{i}$ is the volume fraction of the *i*th filler. Table 2 is a summary of the compositions of the two SM^2^C formulations in this study and one in [22] and their calculated and measured *B_sat_* values. The calculations match well with the measurements within an error 5%.

### 3.4. DC Bias Permeability

**Figure 5:**
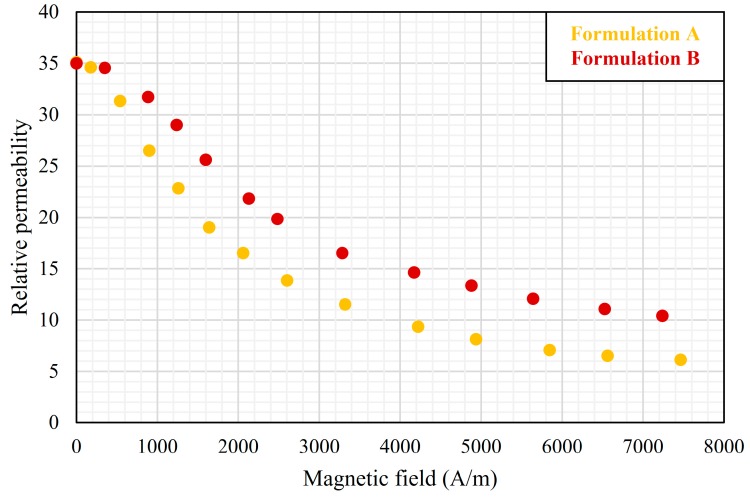
Plots of relative permeability versus DC magnetic field of Formulations A and B.

Figure 5 shows the dependence of the relative permeabilities of our two SM^2^Cs at 10 kHz on the DC bias magnetic field. In both, the relative permeability decreased with increasing bias. The decrease can be explained by the magnetic saturation of the various filler components in the composite. Given the large sizes of the sintered NiZn ferrite flakes used in the composites, initially they served as short-circuit paths for magnetic flux and bear higher magnetic flux density. Since the ferrites also have a lower *B_sat_* than Permalloy, they were easily saturated at low levels of bias. The permeability of Formulation A decreased faster than that of Formulation B because A had more NiZn ferrite flakes in the total magnetic content. After all, the ferrite flakes were saturated, Permalloy particles were left to respond to magnetic field, and the decrease of permeability with DC bias became slower because Permalloy has a larger *B_sat_*.

### 3.5. Microstructure

**Figure 6:**
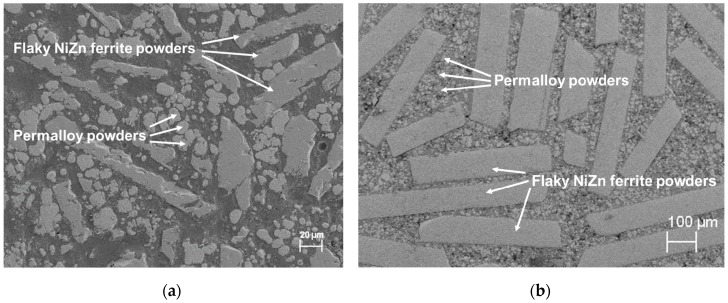
Cross-sectional SEM images of SM^2^Cs (**a**) Formulation A and (**b**) Formulation B.

Figure 6 shows the cross-sectional SEM images of Formulations A and B after curing. The images show magnetic powders embedded in a polymer matrix. The flaky NiZn ferrite powders are randomly distributed in the polymer matrix and the anomalous spherical Permalloy powders filled in the voids between flakes. The high relative permeabilities of our SM^2^Cs were achieved because the flakes served as short-circuit paths for magnetic flux, and the combination of different filler shapes increased the packing density of the magnetic phase. Although the magnetic flux density in the flakes is high, which may increase the hysteresis loss, the high electrical resistivity of the ferrite flakes lowers the eddy-current loss at high frequencies. Therefore, the core-loss densities of our SM^2^Cs made in this study are lower than that from a formulation in [22] and are comparable to those of commercial iron-powder cores. We believe that the magnetic properties of our SM^2^Cs can be further improved by aligning the flaky particles along the direction of magnetic flux through the use of either a mechanical shear force or an external magnetic field [31,32].

## 4. Conclusions

Soft magnetic moldable composites (SM^2^Cs) were formulated by combining Permalloy powders and sintered NiZn ferrite flakes as magnetic fillers in an acrylic polymer. Magnetic cores were fabricated by molding the as-prepared formulations at <200 °C without any external pressure for the potential of forming any core geometries by an injection molding process. The fabricated cores had relative permeabilities over 35 and core-loss densities comparable to, or 30% lower than that of a commercial iron-powder core with the same relative permeability, but required hot-pressing to produce. The advantages of our formulation strategy for ease of core fabrication and good magnetic properties encourage more innovative magnetic component designs and open the door for heterogeneous integration of magnetic components to further improve the power density and efficiency of power converters.

## Figures and Tables

**Table 1:** Compositions of Formulations A and B.

Formula	Formulation A			Formulation B		
Ingredients	NiZn Ferrite Flakes from Powdertech	Permalloy	Binder	NiZn Ferrite Flakes from 3M	Permalloy	Binder
Weight fraction	47.5%	47.5%	5.0%	28.5%	66.5%	5.0%
Mass density (g/cm^3^)	5.20	8.72	1.20	5.20	8.72	1.20
Volume fraction	49%	29%	22%	32%	44%	24%

**Table 2:** Compositions, calculated, and measured saturation magnetic flux density (*B_sat_*) of the Formulations A, B, and in [22].

Formula	Formulation A			Formulation B			Formulation in [22]		
Ingredients	NiZn Ferrite Flakes From Powdertech	Permalloy	Binder	NiZn Ferrite Flakes From 3M	Permalloy	Binder	Metglas Flakes	Permalloy	Binder
Volume fraction	49%	29%	22%	32%	44%	24%	10%	63%	27%
Bsat of raw materials (T)	0.30	0.75	0	0.30	0.75	0	0.77	0.75	0
Calculated Bsat of composite (T)	0.360			0.494			0.570		
Measured Bsat of composite (T)	0.364			0.484			0.547		
Error	4.3%			−1.1%			2.0%		
